# The Use of Headspace Solid-Phase Microextraction (HS-SPME) to Assess the Quality and Stability of Fruit Products: An Example Using Red Mombin Pulp (*Spondias purpurea* L.)

**DOI:** 10.3390/molecules191016851

**Published:** 2014-10-21

**Authors:** Katieli Martins Todisco, Victor Costa Castro-Alves, Deborah dos Santos Garruti, José Maria Correia da Costa, Edmar Clemente

**Affiliations:** 1 Department of Food Technology and Engineering, State University of São Paulo, Rua Cristovão Colombo, 2265, São José do Rio Preto, SP 15054-000, Brazil; E-Mail: katielimar@hotmail.com; 2 Department of Pharmacy, Federal University of Ceará, Rua Alexandre Baraúna, 949, Fortaleza, CE 60430-160, Brazil; E-Mail: vccalves@gmail.com; 3 Laboratory of Sensory Analysis, Embrapa Tropical Agroindustry, Av. Dra. Sara Mesquita, 2270, CP 3761, Fortaleza, CE 60511-110, Brazil; 4 Department of Food Technology, Federal University of Ceará, Av. Mister Hull, 2977, Bloco 858, Fortaleza, CE 60356-000, Brazil; E-Mail: correia@ufc.br; 5 Department of Chemistry, State University of Maringá, Avenida Colombo, 5790, Maringá, PR 87020-900, Brazil; E-Mail: eclemente@uem.br

**Keywords:** volatiles profile, headspace solid-phase microextraction, spray-drier, gas chromatography, fruit powder

## Abstract

The present study aimed to evaluate the volatiles profile of red mombin (*Spondias purpurea*) pulp and its powder produced by spray-drying (SD) as an example to show utility of headspace solid-phase microextraction (HS-SPME) in the analysis of parameters such as the quality and stability of fruit products. Volatiles profiles of the pulp were identified by gas chromatography-mass spectrometry (GC-MS), quantified by gas chromatography-flame ionization detector (GC-FID) and compared to the profile of the powder stored at 0, 60 and 120 days in plastic (PP) or laminated packages (LP). The results showed that the technique was able to identify 36 compounds in the red mombin pulp, 17 out of which have been described for the first time in this fruit, showing that red mombin fresh pulp appears to be unique in terms of volatiles composition. However, only 24 compounds were detected in the powder. This decrease is highly correlated (r^2^ = 0.99), at least for the majority of compounds, to the degree of volatility of compounds. Furthermore, the powder stored in PP or LP showed no statistical differences in the amounts of its components for a period of 120 days of storage. Finally, this work shows how HS-SPME analysis can be a valuable tool to assess the quality and stability of fruit products.

## 1. Introduction

Brazil is a country that stands out for its climatic conditions and a large diversity of tropical fruits with distinctive exotic flavors, which are appealing to foreign consumers [1]. Red mombin (*Spondias purpurea*) is a small red fruit with pleasant aroma and flavor, commonly found in Central America and the Northeast of Brazil [2,3] that is almost exclusively consumed locally due to its relatively short harvest period (between December and January). Nevertheless, this fruit presents good perspectives for commercial purposes since the use of postharvest technologies could extend its shelf life. Derivative products like red mombin powder can add value to this product, increasing the supply and allowing commercialization in regions were the climate is not favorable for its cultivation.

However, fruit processing can affect the flavor, a critical quality attribute for consumers’ acceptance [4,5]. The volatile compounds that are involved in fruit aroma and flavor are produced by several metabolic pathways during ripening, harvest, postharvest and storage periods. Many volatile compounds can be found in a single fruit, but generally only a small group of compounds are responsible for the original aroma of the fruit. The different proportions of these compounds often determine the flavor properties and thus the quality of a fruit [6,7].

Drying is an alternative method to preserve food quality. Reduction of water content and moisture can reduce microbial growth and enzymatic activity, increasing the product’s shelf life [8]. This process is also useful to reduce the product weight and volume, facilitating transportation and handling. One of the most common techniques to remove the water content from fruits is the spray-drying (SD) process that allows food products to dry in a relatively short time and can be carried out on an industrial scale [9,10,11]. One disadvantage is that SD uses heat, which can affect the volatiles composition of the final product. Furthermore, although the powder produced by SD has a longer shelf life than the whole fruit, this process must be evaluated with regard to the sensory attributes of fruits during storage. The classical approach to flavor analysis in food products involves the isolation of volatiles from the matrix, followed by pre-concentration, separation and identification. Headspace solid-phase microextraction (HS-SPME) is a versatile technique for sample preparation and analysis, which offers several advantages such as high sensitivity, good reproducibility, and a simple, quick, solvent-free preparation [12,13]. Among the several uses of HS-SPME, this technique can be a valuable tool for evaluating the processing effect of the volatiles composition of a food product and the stability of volatiles during storage.

In this work, the volatile compounds of red monbim pulp were analyzed in fresh pulp and after the dehydration by the SD process. The stability of the powder volatiles profile after 60 and 120 days of storage in different packages was also checked. The results showed that HS-SPME could be a useful tool in industry to evaluate food products. It was also observed that the volatiles profile of red monbim pulp is stable for a period of longer than 100 days after dehydration. To our knowledge, this is the first time that an attribute related to a red monbim pulp product was evaluated for storage time.

## 2. Results and Discussion

### 2.1. Volatile Composition of Red Mombin Pulp

In this work the HS-SPME technique was used as a tool to assess the quality and stability of red mombin pulp and the powder produced by SD. The use of HS-SPME and subsequent analysis by gas chromatography coupled with mass spectrometry (GC-MS) was able to detect a total of 36 volatile compounds in the headspace of mombim pulp samples, 17 (47.2%) of which have been reported for the first time in this fruit. Thirty-four (94.4%) compounds were identified (Table 1), mostly terpenes (11), followed by alcohols (nine), alkanes (five), esters (five) and ketones (two). 

**Table 1:** Volatile compounds of red mombin (*Spondias purpurea*) pulp and powder stored at 0, 60 or 120 days in plastic (PP) or laminated packages (LP). Each value represents the mean ± SD (×10^6^ chromatogram units) of three analyses of different pools.

Peak	Compound	RI	Integral pulp	Powder				
				After SD	PP		LP	
				(day 0)	(day 60)	(day 120)	(day 60)	(day 120)
1	hexane *	>700	10.7 ± 1.3	1.2 ± 0.1	1.6 ± 0.3	1.1 ± 0.3	1.6 ± 0.5	1.3 ± 0.4
2	ethyl acetate ^a^	>700	107.5 ± 8.4	nd	nd	nd	nd	nd
3	2,3-pentanedione *	>700	41.6 ± 4.3	nd	nd	nd	nd	nd
4	3,3-dimethyl pentane *	>700	11.17 ± 0.47	nd	nd	nd	nd	nd
5	ciclohexane *	>700	6.44 ± 0.12	nd	nd	nd	nd	nd
6	2-methyl-butanol *	>700	4.01 ± 0.31	nd	nd	nd	nd	nd
7	3,4-dimethyl-pentanol *	>700	4.31 ± 0.35	nd	nd	nd	nd	nd
8	1-penten-3-ol *	>700	27.9 ± 6.6	nd	nd	nd	nd	nd
9	2-penten-1-ol *	793	20.1 ± 1.7	2.0 ± 0.7	1.7 ± 0.3	1.9 ± 0.5	1.2 ± 0.1	1.5 ± 0.3
10	2,3-butanediol *	794	18.2 ± 0.5	0.9 ± 0.3	1.0 ± 0.3	0.7 ± 0.1	0.6 ± 0.0	0.7 ± 0.1
11	5-hexen-2-one *	795	1.6 ± 0.1	0.9 ± 0.2	0.7 ± 0.0	0.9 ± 0.4	0.8 ± 0.1	0.7 ± 0.2
12	isopentyl acetate ^b^	795	4.2 ± 0.1	nd	nd	nd	nd	nd
13	4-pentenal *	796	2.4 ± 0.1	nd	nd	nd	nd	nd
14	butyl ethanoate *	797	1.0 ± 0.0	nd	nd	nd	nd	nd
15	Hexanal ^a^	801	194.2 ± 13.6	57.9 ± 10.3	45.4 ± 1.7	41.6 ± 4.9	34.1 ± 2.3	33.7 ± 3.5
16	trans-2-hexenal ^a^	848	4.5 ± 0.3	2.8 ± 0.8	3.1 ± 1.1	1.9 ± 0.6	1.7 ± 0.2	1.4 ± 0.2
17	ethyl 2-methylbutanoate *	852	4.7 ± 0.5	2.7 ± 0.5	2.0 ± 0.0	2.1 ± 0.8	1.8 ± 0.1	1.8 ± 0.1
18	ethyl 3-methylbutanoate	857	nd	2.2 ± 0.7	2.3 ± 0.7	2.3 ± 0.4	2.2 ± 0.2	1.3 ± 1.0
19	3-hexen-1-ol ^a,b^	858	165.2 ± 16.3	97.5 ± 7.3	88.0 ± 6.1	66.8 ± 9.4	89.3 ± 2.7	90.1 ± 8.1
20	NI	867	21.5 ± 2.3	6.0 ± 2.0	7.1 ± 2.6	6.4 ± 1.2	2.9 ± 0.0	2.7 ± 0.6
21	2,6-dimethyl-1-heptene *	870	36.9 ± 0.9	14.1 ± 2.8	15.5 ± 0.2	19.2 ± 3.9	14.4 ± 2.0	13.9 ± 1.1
22	2-hexen-1-ol ^a^	873	114.7 ± 10.8	66.2 ± 3.8	75.6 ± 7.0	66.9 ± 6.1	71.3 ± 5.0	72.7 ± 4.4
**Peak**	**Compound**	**RI**	**Integral pulp**	**Powder**				
				**After SD**	**After SD**		**After SD**	
				**(day 0)**	**(day 0)**	**(day 0)**	**(day 0)**	**(day 0)**
23	β-pinene ^b^	945	nd	5.8 ± 0.5	11.5 ± 4.21	13.5 ± 5.3	9.3 ± 0.1	9.1 ± 0.3
24	β-myrcene ^b^	991	1.9 ± 0.2	nd	nd	nd	nd	nd
25	Limonene ^a,b^	1030	14.0 ± 0.2	27.3 ± 5.4	31.0 ± 3.0	27.0 ± 5.1	23.5 ± 1.7	24.1 ± 3.3
26	Copaene ^b^	1373	15.0 ± 1.6	nd	nd	nd	nd	nd
27	β-caryophyllene ^a^	1415	45.2 ± 7.3	1.7 ± 0.3	1.6 ± 0.4	1.4 ± 0.4	0.9 ± 0.1	0.9 ± 0.1
28	non identified terpene	1446	2.8 ± 0.5	0.5 ± 0.0	0.5 ± 0.1	0.4 ± 0.1	0.2 ± 0.0	0.4 ± 0.2
29	α-caryophyllene ^a^	1452	14.6 ± 1.5	0.7 ± 0.1	0.5 ± 0.1	0.5 ± 0.1	0.4 ± 0.0	0.4 ± 0.0
30	NI terpene	1470	1.6 ± 0.2	1.2 ± 0.4	0.7 ± 0.0	0.7 ± 0.2	0.6 ± 0.0	0.6 ± 0.1
31	NI terpene	1472	4.3 ± 0.1	0.2 ± 0.1	0.2 ± 0.1	0.2 ± 0.0	0.2 ± 0.0	0.2 ± 0.0
32	α-muurolene *	1494	4.8 ± 0.5	tr	tr	0.4 ± 0.1	0.3 ± 0.0	0.3 ± 0.0
33	pentadecane *	1500	2.5 ± 0.1	2.5 ± 3.7	0.3 ± 0.1	0.4 ± 0.1	0.3 ± 0.0	0.3 ± 0.0
34	NI terpene	1514	8.2 ± 0.4	1.5 ± 2.2	0.3 ± 0.1	0.4 ± 0.1	0.3 ± 0.0	0.3 ± 0.0
35	NI	1615	81.9 ± 3.0	3.3 ± 2.1	4.5 ± 0.9	5.1 ± 1.4	4.8 ± 0.1	5.3 ± 0.9
36	α-bisabolol *	1683	19.2 ± 2.1	0.9 ± 0.2	0.7 ± 0.0	0.6 ± 0.2	0.9 ± 0.1	1.3 ± 0.7

RI: retention index in a DB-5 column; nd: not detected; tr: traces (>0.01 × 10^6^ counts); NI: non identified; *: first time identified in red mombin; ^a^: also identified by Ceva-Antunes* et al.* [14]; ^b^: also identified by Augusto* et al.* [15].

Using the same technique, Augusto* et al.* [15] identified 19 compounds with a carboxen/polydimethylsiloxane (CAR/PDMS) fiber coating (eight esters, seven alcohols, two aldehydes and two ketones). These authors, however, did not specify the relative amount of the compounds found in the chromatograms and used a higher temperature for the volatiles extraction (60 °C), which may have led to the formation of artifacts or even the degradation of some of the aroma compounds. 

In another work with red mombin pulp, Ceva-Antunes* et al.* [14] evaluated the extraction efficiency of volatiles using four types of SPME fiber coatings. The authors showed that the divinylbenzene/carboxen/polydimethylsiloxane (DVB/CAR/PDMS) fiber, used in the present work, extracted a larger number (27) and amount of volatile compounds from red mombin pulp. They identified nine esters, seven aldehydes, five terpenes, four alcohols and two ketones. This was an important study since several coatings of SPME fibers are available for the extraction of compounds with different levels of polarity, but no one will extract all volatiles at the same extent. Therefore, for each type of sample, it is important to evaluate fibers of different polarities.

The differences between the red mombin volatile profiles determined by Ceva-Antunes* et al.* [14] and the present work should be related to: (1) the sampling method; (2) the harvest season; or even (3) environmental factors, such as climate and soil, which can have an effect on the volatile metabolic pathways [16,17,18,19]. In this work it was used a relatively large sample (20 kg) derived from distinct regions and climatic conditions from different regions of Brazilian Northeast. Thus, the sampling allowed the identification of more compounds when compared to studies that evaluated fruits from specific regions [14].

**Figure 1:**
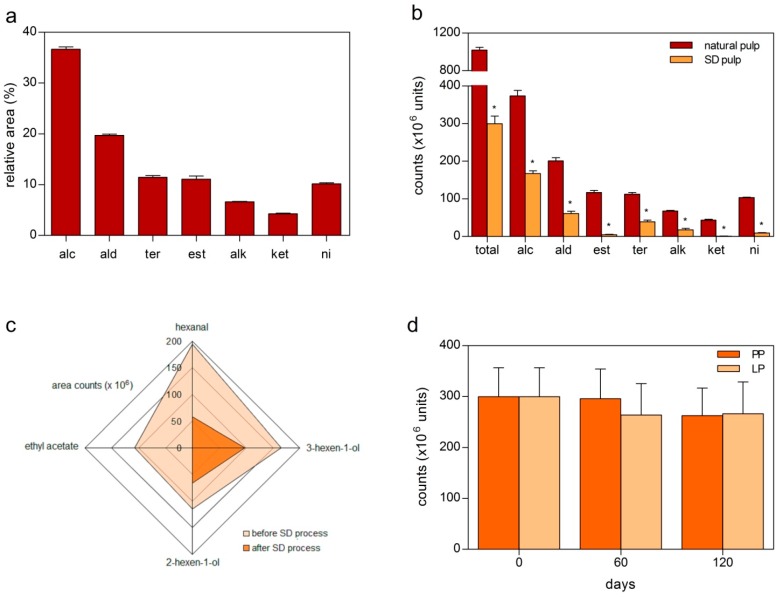
(**a**) Relative chromatogram area of alcohols (alc), aldehydes (ald), terpenes (ter), esters (est), alkanes (alk), ketones (ket) and non-identified (ni) compounds in red mombin pulp; (**b**) chromatogram area of red mombin pulp before and after the spray-drying (SD) process; (**c**) chromatogram area of the majority of compounds of the red mombin pulp before and after the SD process; (**d**) chromatogram area of volatile compounds of the powder of red mombin pulp during storage with plastic (PP) or laminated package (LP). Values represent the mean of three analyses. *: significantly difference (*p* < 0.001; *t* test).

In order to analyze the volatile compounds of natural red mombin pulp, we performed three extractions using different pools of pulp. The results showed that the total chromatograms areas were quite similar among extractions, with a coefficient of variation of 4.92%. The same compounds were identified in the extractions, with the exception of butyl ethanoate, which was detected only in one injection, as the compound present in the lowest amount (representing only 0.08% of the chromatogram relative area). Regarding peak area, alcohols were the major class of fresh red mombin pulp volatiles, followed by aldehydes, esters and terpenic compounds (Figure 1a). 

Although these results are different from another study [14] that found esters and aldehydes as the main chemical classes in the red mombin fruit, most compounds are almost the same. In our work, we found hexanal, 3-hexen-1-ol, 2-hexen-1-ol and ethyl acetate as the major compounds, while Ceva-Antunes* et al.* [14] found hexanal, 3-hexen-1-ol, trans-2-hexenal, 2-hexen-1-ol, hexyl acetate and ethyl acetate. Terpenes were more abundant in number of compounds, differing from other tropical fruits, including species of *Spondias* genre like umbu-caja (*S. citherea*), taperebá (*S. mombin*) and cajá (*S. mombin*) [20,21]. *Spondias purpurea*, therefore, it appears to be unique in terms of volatile composition.

### 2.2. Effects of Spray-Drying (SD) on the Volatiles Composition of Red Mombin Pulp

The volatiles profile of red mombin pulp dehydrated by the SD process was also evaluated. In order to allow a comparison between the integral pulp and the dry material, the powder was reconstituted in distilled water until it reached the same amount of soluble solids of the pulp before the dehydration (17 °Brix). Results showed a significantly difference between the volatile profiles of the pulp before and after the SD process, as can be seen in the total chromatogram areas (Table 1). The amount of volatiles decreased 70.56% after the SD process, especially for the ketones and esters, which showed a reduction of chromatogram area of 98.04% and 95.81%, respectively (Figure 1b). 

With regard to the major volatile compounds of red mombin pulp, the losses during the drying process were highly correlated (r^2^ = 0.99; Pearson correlation) with the degree of volatility of these compounds. Ethyl acetate, a relatively highly volatile compound (T_boiling_ = 77.2 °C) was not observed after the SD process, while hexanal (T_boiling_ = 129.0 °C) suffered a loss of approximately 70% and the alcohols 2-hexen-1-ol (T_boiling_ = 159.0 °C) and 3-hexen-1-ol (T_boiling_ = 157.0 °C) lost 41% and 42%, respectively (Figure 1c).

Only 24 out of 36 compounds detected in the pulp remained in the dried product and only limonene (peak 25; Table 1) was detected in larger amounts after drying. Two other compounds were identified only in the processed product: ethyl-3-methylbutanoate (peak 18; Table 1) and the β-pinene (peak 23; Table 1). Although the amounts of the majority of the volatile compounds detected in the pulp decreased after the SD process, the use of a high temperature (120 °C) could promote a disruption of the fresh pulp cells, modifying the molecular interactions between flavor compounds and matrix constituents [22] and releasing products that were bound to the matrix. This could explain the higher amounts of limonene and the appearance of new volatile compounds after the SD process.

The results showed that, as already observed for roselle extracts (*Hibiscus sabdariffa* L.) [23], only part of the volatile compounds from the pulp is retained in the powder after the SD process. The degradation induced by the heat can lead the formation of substances which were not previously detected, suggesting that SD may have a significant effect on the qualitative and quantitative composition of red mombin pulp and possibly in their acceptance by the consumer. Further studies are needed to assess the effects of this processing method in the sensory quality of the product.

### 2.3. Volatile Compound Stability of the Red Mombin Pulp Stored in Different Packages

The drying process tends to facilitate transportation of the material in addition to providing greater stability, reducing the growth of microorganisms and the physical and chemical degradation of substances. When the product is properly packed, oxygen contact and moisture gain are avoided, thus preventing agglomeration and solidification, which may give a longer shelf life to the product [24]. Based on these advantages, we decided to evaluate the volatiles composition of the powder stored for 60 and 120 days in plastic packages (PP) or laminated packages (LP), subjected to storage conditions of 25 ± 2 °C and 85% ± 5% of relative humidity. The results showed that both packages were able to maintain the volatile composition of the powder for the studied storage period, as can be seen in the total chromatogram area of the volatile profiles (Figure 1d). Thus, LP or PP could be adequate for the maintenance of the volatile composition of the product for a relatively long period of storage.

## 3. Experimental Section

### 3.1. Materials

Frozen red mombin (*Spondias purpurea*) pulp was acquired from the Ki-Polpa company (Fortaleza, CE, Brazil) and transported to the Laboratory of Quality Control and Drying of the Department of Food Technology at Federal University of Ceará. The pulp was whole, free of added water, with conserving and thermal treatments. Samples (n = 200) from the same batch (number 1, 05196-00017-5) in packages of 100 g were used for analyses. The material was stored at −18 °C until processing.

The red mombin pulp powder was obtained by drying a solution containing 90% of integral pulp and 10% of maltodextrin (DE = 20) in a LM MSD 1.0 spray-dryer (SD, Labmaq, Ribeirão Preto, SP, Brazil) with an inlet drying temperature of 120 °C, outlet temperature of 80 °C and flow rate of 240 mL·h^−1^. Samples of pulp powder (10 g) were packed in plastic (PP: pet-ethylene polyterephthalate + polypropylene/pet + polyethylene film, density of 100 g∙m^−2^) or laminated packages (LP: aluminium/pet + ADES + aluminium/ADES + polyethylene film, density of 122 g∙m^−2^), sealed and stored for 120 days at 25 ± 2 °C and 85% ± 5% of relative humidity.

### 3.2. Headspace Extraction Procedure

The volatile compounds were extracted according to the HS-SPME methodology optimized by Ceva Antunes* et al.* [14], using 6 g of natural red mombin pulp in 40 mL amber glass vials with polytetrafluoroethylene/silicone septa. The powder was reconstituted in distilled water until 17 °Brix, the same pulp soluble solids content before the dehydration. In each sample was added NaCl 30% (w/v) to reduce the solubility of organic compounds and increase the volatile extraction. DVB/CAR/PDMS (50/30 µm f.t.) fibers (Supelco, Bellefonte, PA, USA) were previously conditioned according to the manufacturer’s instructions and then exposed to the vial’s headspace under the following conditions: extraction time of 60 min at 25 °C, magnetic stirring at 250 rpm.

### 3.3. Gas Chromatography-Flame Ionization Detector (GC-FID) Analyses

The GC-FID analyses were adapted from a previously study [14] and carried out using a GC CP-3380 instrument (Agilent, Palo Alto, CA, USA) equipped with a CP-Sil 8 CB (5% phenyl-polymethylsiloxane; 30 m × 0.25 mm i.d.; 0.25 µm f.t.) capillary column (Agilent). After the extraction of volatile compounds from the HS, the fiber was injected into the GC-FID in the splitless mode for 2 min. The injector temperature was kept at 250 °C and the detector at 280 °C. Hydrogen was used as the carrier gas at a constant flow of 1.5 mL·min^−1^. The oven temperature programming started at 40 °C (2 min) and increased at 3 °C·min^−1^ to 210 °C (5 min). Analyses were performed in triplicates for each sample.

### 3.4. Gas Chromatography-Mass Spectrometry (GC-MS) Analyses

Identification of volatile compounds was performed in a GC-2010 instrument (Shimadzu, Kyoto, Japan) equipped with a QP-2010 mass spectrometer and DB-5 MS (30 m × 0.25 mm i.d.; 0.25 µm f.t.) capillary column (Agilent). The carrier gas was helium at a constant flow of 1.0 mL·min^−1^. Injection conditions, detector temperature and oven temperature gradient were the same used for GC-FID analyses. Compounds were identified by comparing the mass spectra with those provided by the library of the National Institute of Standards and Technology (NIST) [25]. Retention indices (Kovats index) were calculated from a homologous series of alkanes (C_7_–C_21_). Peaks that showed similarity lower to 90% when compared to the spectrum or retention indices different to the library reference were excluded from analysis. Compounds were considered tentatively identified when it was based only on mass spectral data.

## 4. Conclusions

This study has showed that *Spondias purpurea* pulp appears to be unique in terms of volatiles composition. Furthermore, it was demonstrated that despite the significant reduction in volatile compounds after the SD process, the powder produced seems to be stable regarding its volatiles composition since the chromatogram area of dried samples stored in PP or LP showed no statistical differences among them after a period of 120 days of storage. It was possible to verify that the product could have sufficient stability to be marketed. However, more studies are needed to verify its nutritional aspects, as well consumer acceptance. Finally, this work shows how HS-SPME analysis can be a valuable tool to assess the quality and stability of fruit products.

## References

[1] Genovese M.I., Pinto M.S., Gonçalves A.E.S., Lajolo F.M. (2008). Bioactive compounds and antioxidant capacity of exotic fruits and commercial frozen pulps from Brazil. Food Sci. Technol. Int..

[2] Narain N., Galvão M.S., Santana K.L., Moreira J.J.S. (2010). Volatile compounds in tomato-based dried products. Dry. Technol..

[3] Augusto P.E.D., Cristianini M., Ibarz A. (2012). Effect of temperature on dynamic and steady-state shear rheological properties of siriguela (*Spondias purpurea* L.) pulp. J. Food Eng..

[4] Nardini G.S., Merib J.O., Dias A.N., Dutra J.N.B., Silveira C.D.S., Budziak D., Matrendal E., Carasek E. (2013). Determination of volatile profile of citrus fruit by HS-SPME/GC-MS with oxidized NiTi fibers using two temperatures in the same extraction procedure. Microchem. J..

[5] Gokbulut I., Karabulut I. (2012). SPME–GC–MS detection of volatile compounds in apricot varieties. Food Chem..

[6] Torres J.D., Chiralt A., Escriche I. (2012). Development of volatile fraction of fresh cut osmotically treated mango during cold storage. Food Chem..

[7] Sunthonvit N., Srzednicki G., Craske J. (2007). Effects of drying treatments on the composition of volatile compounds in dried nectarines. Dry. Technol..

[8] Thuwapanichayanan R., Prachayawarakorn S., Kunwisawa J., Soponronnarit S. (2011). Determination of effective moisture diffusivity and assessment of quality attributes of banana slices during drying. LWT Food Sci. Technol..

[9] Zotarelli M.F., Porciuncula B.D.A., Laurindo J.B. (2012). A convective multi-flash drying process for producing dehydrated crispy fruits. J. Food Eng..

[10] Demarchi S.M., Ruiz N.A.Q., Concellón A., Giner S.A. (2013). Effect of temperature on hot-air drying rate and on retention of antioxidant capacity in apple leathers. Food Bioprod. Process..

[11] Borrmann D., Pierucci A.P.T.R., Leite S.G.F., Leão M.H.M.R. (2013). Microencapsulation of passion fruit (*Passiflora*) juice with n-octenylsuccinate-derivatised starch using spray-drying. Food Bioprod. Process..

[12] Cheong K.W., Tan C.P., Mirhosseini H., Hamid N.S.A., Osman A., Basri M. (2010). Equilibrium headspace analysis of volatile flavor compounds extracted from soursop (*Annona muricata*) using solid-phase microextraction. Food Res. Int..

[13] Dong L., Piao Y., Zhang X., Zhao C., Hou Y., Shi Z. (2013). Analysis of volatile compounds from a malting process using headspace solid-phase micro-extraction and GC-MS. Food Res. Int..

[14] Ceva-Antunes P.M.N., Bizzo H.R., Silva A.S., Carvalho C.P.S., Antunes O.A.C. (2006). Analysis of volatile composition of red mombin (*Spondias purpurea* L.) by solid phase microextraction (SPME). LWT Food Sci. Technol..

[15] Augusto F., Valente A.L.P., Tada E.S., Rivellino S.R. (2000). Screening of Brazilian fruits aromas using solid-phase microextraction-gas chromatography-mass spectrometry. J. Chromatogr. A.

[16] Watson R., Wright C.J., McBurney T., Taylor A.J., Linforth R.S.T. (2002). Influence of harvest date and light integral on the development of strawberry flavour compounds. J. Exp. Bot..

[17] Yu-Tao W., Shao-Wen H., Rong-Le L., Ji-Yun J. (2007). Effects of nitrogen application on flavor compounds of cherry tomato fruits. J. Plant Nutr. Soil Sci..

[18] Song J., Fan L., Forney C.F., Jordan M.A. (2001). Using volatile emission and chlorophyll fluorescence as indicators of heat injury in apples. J. Am. Soc. Hortic. Sci..

[19] Hewett E.W. (2006). An overview of preharvest factors influencing postharvest quality of horticultural products. Int. J. Postharvest Technol. Innov..

[20] Ceva-Antunes P.M.N., Bizzo H.R., Alves S.M., Antunes O.A.C. (2003). Analysis of volatile compounds of tapereba (*Spondias mombin* L.) and caja (*Spondias mombin* L.) by simultaneous distillation and extraction (SDE) and solid phase microextraction (SPME). J. Agric. Food Chem..

[21] Franco M.R.B., Shibamoto T. (2000). Volatile composition of some brazilian fruits: Umbu-caja (*Spondias citherea*), camu-camu (*Myrciaria dubia*), Araça-boi (*Eugenia stipitata*), and cupuaçu (*Theobroma grandi**florum*). J. Agric. Food Chem..

[22] Secouard S., Maliac C., Grisel M., Decroix B. (2003). Release of limonene from polysaccharide matrices: Viscosity and synergy effect. Food Chem..

[23] Gonzalez-Palomares S., Estarrón-Espinoza M., Gómez-Leyva J.F., Andrade-González I. (2009). Effect of the temperature on the spray drying of roselle extracts (*Hibiscus sabdariffa* L.). Plant Foods Hum. Nutr..

[24] Tze N.G., Han C.P., Yusof Y.A., Ling C.N., Talib R.A., Taip F.S., Aziz M.G. (2012). Physiochemical and nutritional properties of spray-dried pitaya fruit powder as natural colorant. Food Sci. Biotechnol..

[25] NIST, National Institute of Standards and Technology Database Standard Reference Number 69. http://webbook.nist.gov/chemistry/.

